# Lgr5^+^ amacrine cells possess regenerative potential in the retina of adult mice

**DOI:** 10.1111/acel.12346

**Published:** 2015-05-20

**Authors:** Mengfei Chen, Shenghe Tian, Nathan G Glasgow, Gregory Gibson, Xiaoling Yang, Christen E Shiber, James Funderburgh, Simon Watkins, Jon W Johnson, Joel S Schuman, Hongjun Liu

**Affiliations:** 1 Department of Microbiology and Molecular Genetics, University of Pittsburgh School of MedicinePittsburgh, PA, USA; 2 Department of Ophthalmology and Visual Science Research Center, University of Pittsburgh School of MedicinePittsburgh, PA, USA; 3 Louis J. Fox Center for Vision Restoration of UPMC and the University of PittsburghPittsburgh, PA, USA; 4 Department of Neuroscience and Center for Neuroscience, University of PittsburghPittsburgh, PA, USA; 5 Center for Biologic Imaging, University of Pittsburgh School of MedicinePittsburgh, PA, USA; 6 UPMC Eye Center, Eye and Ear InstitutePittsburgh, PA, USA; 7 Department of Cell Biology and Physiology, University of Pittsburgh School of MedicinePittsburgh, PA, USA; 8 Department of Bioengineering, Swanson School of Engineering, University of PittsburghPittsburgh, PA, USA

**Keywords:** aging, amacrine cells, Lgr5, retina, neurogenesis, retinal regeneration

## Abstract

Current knowledge indicates that the adult mammalian retina lacks regenerative capacity. Here, we show that the adult stem cell marker, leucine-rich repeat-containing G-protein-coupled receptor 5 (Lgr5), is expressed in the retina of adult mice. Lgr5^+^ cells are generated at late stages of retinal development and exhibit properties of differentiated amacrine interneurons (amacrine cells). Nevertheless, Lgr5^+^ amacrine cells contribute to regeneration of new retinal cells in the adult stage. The generation of new retinal cells, including retinal neurons and Müller glia from Lgr5^+^ amacrine cells, begins in early adulthood and continues as the animal ages. Together, these findings suggest that the mammalian retina is not devoid of regeneration as previously thought. It is rather dynamic, and Lgr5^+^ amacrine cells function as an endogenous regenerative source. The identification of such cells in the mammalian retina may provide new insights into neuronal regeneration and point to therapeutic opportunities for age-related retinal degenerative diseases.

## Introduction

The mammalian retina is a well-characterized structure consisting of six types of neurons and one type of glia. These neurons and glial cells are arranged into a stereotypical laminar organization to facilitate the translation of light stimuli captured by photoreceptors into electrical signals that are then transmitted to the brain by ganglion neurons (Masland, 2001). All of the neurons and glial cells of the mature retina are derived from a population of multipotent progenitor cells during retinal histogenesis (Livesey & Cepko, 2001). In lower vertebrates, such as fish and amphibians, a population of progenitor cells located at the margin of the retina, the ciliary marginal zone, continuously proliferates and differentiates, thereby adding new cells to the periphery of the existing retina throughout the organism’s lifetime (Amato *et al*., 2004; Moshiri *et al*., 2004; Lamba *et al*., 2008). The ciliary marginal zone is greatly reduced in birds and absent in mammals, consistent with the limited avian retinal proliferative capacity and the presumed absence of neurogenesis in the retina of adult mammals (Kubota *et al*., 2002). This absence of regeneration is thought to contribute to a host of retinal degenerative diseases, including age-related macular degeneration, glaucoma and retinitis pigmentosa, which have been viewed as consequences of the irreversible loss of retinal neurons (Rattner & Nathans, 2006; Bhatia *et al*., 2010; Moore & Goldberg, 2010; Wohl *et al*., 2012).

Given that retinal stem cells hold great therapeutic potential for vision restoration in people who suffer from blindness associated with retinal degeneration, numerous attempts have been made to identify these cells in adult mammals. Nearly a decade ago, a population of pigmented cells was isolated from the ciliary body in mice, a region between the retina and the iris (Ahmad *et al*., 2000; Tropepe *et al*., 2000). Initial observations suggested this cell population possesses retinal stem cell properties, yet further analysis demonstrated that these cells could not differentiate into retinal neurons *in vitro* or *in vivo* (Cicero *et al*., 2009; Gualdoni *et al*., 2010). Based on the observation that neural stem cells found in regions of the adult brain possess glial properties (Doetsch *et al*., 1999; Seri *et al*., 2001), subsequent studies have focused on the regenerative potential of the retinal Müller glial cell (Dyer & Cepko, 2000; Fischer & Reh, 2001; Ooto *et al*., 2004; Karl *et al*., 2008). Some studies have documented that this cell type can proliferate and give rise to other cell types in response to injury (Osakada *et al*., 2007; Karl *et al*., 2008). Nonetheless, Müller glial cells in the retina do not proliferate under normal physiological conditions and their potential to generate other retinal cell types after injury is also limited (Karl *et al*., 2008). Therefore, whether the retina in adult mammals possesses regenerative capacity under normal physiological conditions still remains undetermined and, if so, the cellular source that serves as the precursors for retinal regeneration in adulthood is also unknown.

Here, we demonstrate that the adult stem cell marker Lgr5 is expressed in a subset of inner nuclear layer retinal cells in the eye of adult mice. These Lgr5^+^ retinal cells are derived from proliferating retinal progenitor cells at late stages of retinogenesis and can be categorized as amacrine interneurons. Although they exhibit properties of differentiated interneurons, they are able to contribute to retinal regeneration. Newly generated cells exhibit features of both retinal neurons and Müller glia. The generation of new retinal cells by Lgr5^+^ amacrine cells in adult mice begins in early adulthood and continues with advancing age. Together, these results suggest that the retina in adult mammals is not devoid of regeneration as previously thought; rather, it is dynamic. Lgr5^+^ amacrine cells may function as an endogenous regenerative source and contribute to continuous retinal regeneration throughout life.

## Results

### Lgr5 marks a subset of retinal amacrine cells in adult mice

**Fig 1:**
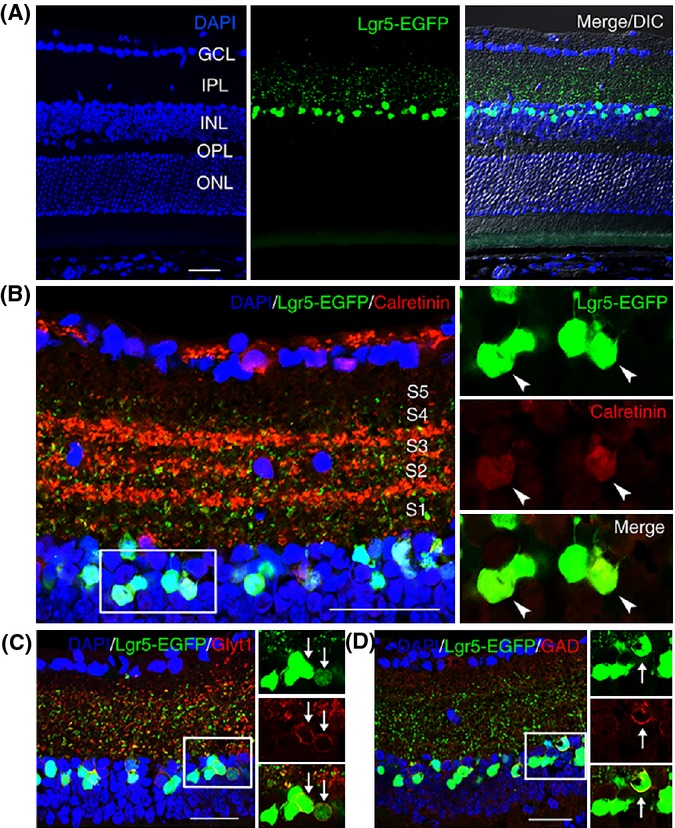
Lgr5-EGFP marks a subset of amacrine cells in the inner nuclear layer of adult mouse retina. (A) Confocal images of EGFP expression from the *Lgr5* locus (Lgr5-EGFP) in the retina of 8-week-old *Lgr5*^*EGFP*^^*-Ires-Cre*^^*ERT*^^*2*^ mice. Lgr5-EGFP^+^ cells are restricted to the inner nuclear layer with axons projecting to the inner plexiform layer. (B) Staining of *Lgr5*^*EGFP*^^*-Ires-Cre*^^*ERT*^^*2*^ mouse retina with anti-calretinin antibody. The majority of Lgr5-EGFP^+^ cells are also positive for calretinin. The processes of Lgr5-EGFP^+^ cells can reach sublaminal layer 5 (S5) within the inner plexiform layer. (C) Staining of *Lgr5*^*EGFP*^^*-Ires-Cre*^^*ERT*^^*2*^ mouse retina with anti-GlyT1 antibody. (D) Staining of *Lgr5*^*EGFP*^^*-Ires-Cre*^^*ERT*^^*2*^ mouse retina with anti-GAD antibody. Boxed areas in panels (B–D) are highlighted in adjacent higher magnification views, with overlapping signals indicated by arrows or arrowheads. GCL, ganglion cell layer; IPL, inner plexiform layer; INL, inner nuclear layer; OPL, outer plexiform layer; ONL, outer nuclear layer. Scale bars = 30 μm.

The adult stem cell marker Lgr5 belongs to a family of G-protein-coupled receptors (Luo & Hsueh, 2006; van der Flier & Clevers, 2009) and has been used to identify adult stem cells in multiple tissues (Barker *et al*., 2007; Snippert *et al*., 2010; Chai *et al*., 2012; Shi *et al*., 2012; Yee *et al*., 2013; Chen *et al*., 2014). To assess whether Lgr5 is expressed in the mouse retina, we examined the expression of the *Lgr5* gene in the retina of *Lgr5*^*EGFP-Ires-CreERT2*^ knock-in mice, which express EGFP and the inducible Cre recombinase bi-cistronically from the endogenous *Lgr5* locus (Barker *et al*., 2007). We found that EGFP expressed from this locus (hereafter referred to as Lgr5-EGFP) was detected in the adult mouse retina and limited to a population of cells within the inner half of the inner nuclear layer (Fig.1A). The majority of these Lgr5-EGFP^+^ cells were arranged in a ‘zigzag’ pattern occupying the second and third inner cell rows (Fig.1A,B). By contrast, Lgr5-EGFP was not detected in the ciliary body (Fig. S1; Supporting information).

The inner nuclear layer of the retina is composed of four cell types. These consist of horizontal cells, bipolar cells, and amacrine cells, each of which are interneurons that relay electrical signals from photoreceptors to ganglion cells, and Müller cells, which are specialized supportive astrocytes. To identify which cell types expressed Lgr5-EGFP, we used antibodies for cell-specific markers. We found that Lgr5-EGFP co-localized with the amacrine cell markers syntaxin 1A and Pax6, but not with markers of the other cell types (Fig. S2; Supporting information). Characteristic of amacrine cells, Lgr5-EGFP^+^ cells projected axonal processes into the inner plexiform layer and the majority of these cells also expressed calretinin (Fig.1A,B). Amacrine cells are the most diverse interneurons in the retina and can be categorized into approximately 30 subgroups according to their specific morphology. The majority of amacrine cells use either GABA or glycine as a neurotransmitter (MacNeil & Masland, 1998; Hsueh *et al*., 2008). We next asked whether Lgr5-EGFP expression was restricted to GABAergic or glycinergic amacrine cells. We found that Lgr5-EGFP co-localized with either the glycinergic marker glycine membrane transporter 1 (GlyT1) or the GABAergic marker glutamic acid decarboxylase (GAD) (Fig.1C,D). Together, these findings suggest that Lgr5-EGFP is expressed in a subgroup of amacrine interneurons within the inner nuclear layer of the adult mammalian retina.

### Lgr5^+^ amacrine cells are generated at a late stage of retinogenesis

**Fig 2:**
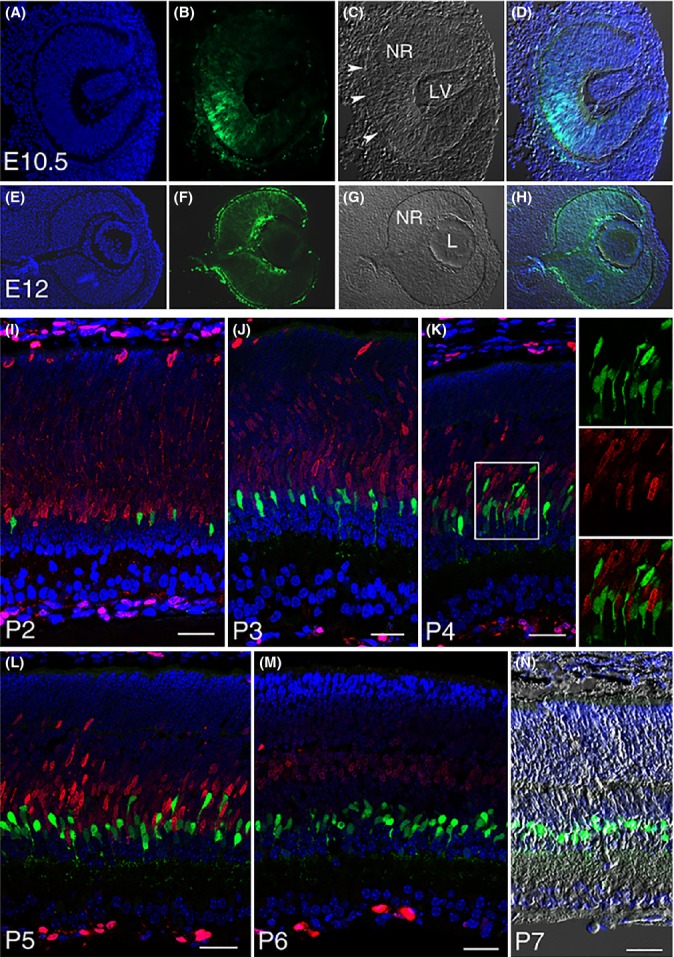
Lgr5-EGFP expression at various stages of retinogenesis. (A–D) Lgr5-EGFP expression in the developing eye is first detected in the optic cup at E10.5, beginning in the middle of the optic cup and then expanding to its peripheral margin. Arrowheads in *C* highlight retinal pigmented epithelium. NR, neural retina; LV, lens vesicle. (E–H) Lgr5-EGFP expression in the neural retina (NR), lens (L), retinal pigmented epithelium, and surrounding mesenchymal cells at E12. (I–M) Confocal images of *Lgr5*^*EGFP*^^*-Ires-Cre*^^*ERT*^^*2*^ mouse retina co-stained with Ki67 (red) at postnatal day 2 through day 6. Lgr5-EGFP^+^ cells do not express Ki67 at any stage of late retinogenesis, indicating that they have exited the cell cycle. Boxed area in panel *K* is highlighted in the adjacent panels allowing for higher magnification views. (N) Merged image of DAPI staining, Lgr5-EGFP, and DIC of a retinal section at P7. In all relevant panels, Lgr5-EGFP is in green and DAPI staining is in blue. Scale bars = 30 μm.

The expression of the Lgr5 stem cell marker in amacrine interneurons was unexpected given the classical perception that neurons represent a developmental endpoint. Therefore, we investigated the possibility that Lgr5-EGFP might label a population of previously unidentified retinal progenitor cells that skipped terminal differentiation at a late stage of retinogenesis and persisted into adulthood. Retinogenesis in mice begins during embryogenesis at embryonic day 10 (E10) to E11 when the optic vesicle invaginates to form the optic cup and is completed by postnatal day 15 (P15) (Pei & Rhodin, 1970). Amacrine cells are generated over a broad temporal window, starting shortly after the optic cup is formed and lasting until P5 to P6 (Voinescu *et al*., 2009). The Lgr5-EGFP signal was detected shortly after optic vesicle invagination on E10.5 in both layers of the optic cup and surrounding mesenchymal cells. Within the optic cup, Lgr5-EGFP expression was first detected in the middle or bottom and then extended in a gradient toward the peripheral margin (Fig.2A–D). By E12, the optic cup cells were uniformly Lgr5-EGFP^+^ and the majority of the Lgr5-EGFP^+^ mesenchymal cells had migrated to the vicinity of the optic cup and into the cavity between the optic cup and the lens vesicle. The lens vesicle also expressed Lgr5-EGFP at this stage (Fig.2E–H).

Embryonic Lgr5-EGFP expression then rapidly declined and was absent by E15 but reappeared postnatally at late P2, when scattered individual Lgr5-EGFP^+^ cells were detected in the inner half of the retina (Fig.2I). More Lgr5-EGFP^+^ retinal cells were observed in the following days. These cells lined up in a row next to the proliferating retinal progenitor cell zone at P3 and projected processes toward the inner plexiform layer at P4 (Fig.2J,K). By P5, the majority of Lgr5-EGFP^+^ retinal cells had migrated to the vitreous side of the retina (Fig.2L). By P6, Lgr5-EGFP^+^ cells were completely separated from the proliferating retinal progenitor cell zone (Fig.2M), and by P7, the majority of these cells had migrated to their final destination in the second and third rows of the inner nuclear layer (Fig.2N). Throughout this period of postnatal retinogenesis, Lgr5-EGFP^+^ cells were negative for Ki67, suggesting that they had exited the cell cycle and differentiated. This conclusion is further supported by the formation of a loosely organized sublaminal structure in the inner plexiform layer after these cells reached the vitreous side of the retina and the subsequent formation of synapse-like contacts before retinogenesis was completed (Fig.2L,M, and Fig. S3; Supporting information). Taken together, these results demonstrate that Lgr5-EGFP is expressed biphasically during retinogenesis and labels a small subset of differentiated amacrine interneurons in postnatal mice that are distinct from late-stage proliferating retinal progenitor cells.

### Generation of other retinal lineages by Lgr5^+^ amacrine cells *in vivo*

**Fig 3:**
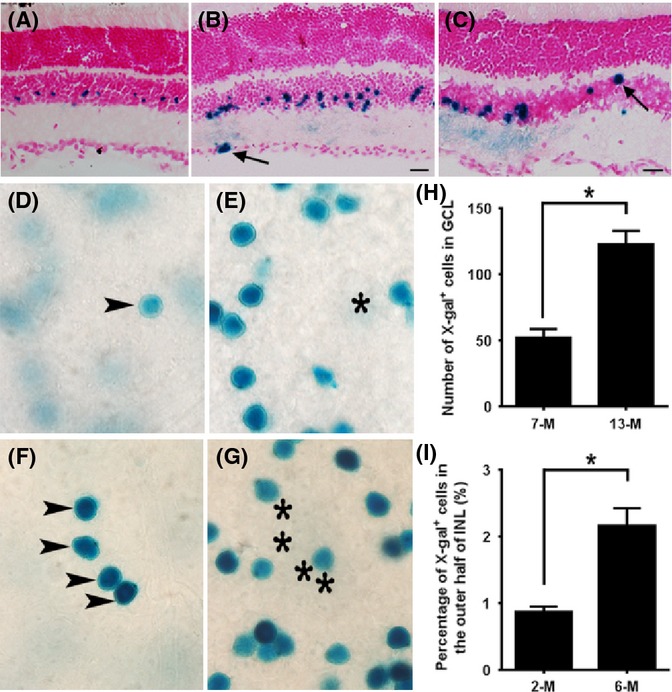
Lineage tracing of Lgr5-EGFP^+^ cells in mouse retinas. (A–C) X-gal staining (blue) of retinas from *Lgr5*^*EGFP*^^*-Ires-Cre*^^*ERT*^^*2*^*; Rosa26-LacZ* mice at 3 weeks (A), 2 months (B), and 6 months (C) of age following tamoxifen injection at P3 to P4 (A, B) or 6 weeks of age (C). Arrows mark LacZ-positive cells in the retinal ganglion layer (B) and outer edge of the inner nuclear layer (C). (D–G) Images of whole-mount retinal samples stained with X-gal following tamoxifen injection at 4–6 weeks of age. Images in panels D and E were taken from the same location of the same retina sample, with D focused to the ganglion cell layer and E focused to the inner nuclear layer. The X-gal^+^ cell located in the ganglion cell layer (D) is highlighted with an arrowhead. Its corresponding vertical location in the inner nuclear layer was marked with a star (E). Similar to panels D and E, X-gal staining images in panels F and G are taken from the same retinal sample at the same location, with panel F focused to the ganglion cell layer and panel G focused to the inner nuclear layer. At this location, four X-gal^+^ cells form a cluster in the ganglion cell layer. (H) Numbers of X-gal^+^ cells in the ganglion cell layer in 7-month-old and 13-month-old mice. *n* = 6 retinas from three mice of each group. *P = 0.004 by Student’s *t*-test. (I) Percentage of X-gal-positive cells that are located in the outer half of the inner nuclear layer in 2-month-old and 6-month-old mice. *n* = 8 and 12 sections from three mice of each group. *P = 0.01 by Student’s *t*-test. Scale bars = 20 μm.

Given that Lgr5^+^ cells function as adult stem cells in multiple tissues and organs, we speculate that these Lgr5-EGFP^+^ amacrine cells might also possess regenerative capacity, although this contradicts the current dogma that neurons represent a developmental endpoint. We reasoned that the adult mammalian retina is a highly specialized tissue that precludes a high cell turnover. Cells that function as a regenerative source may also have other physiological functions, including neuronal activities. To test this hypothesis and to explore the regenerative potential of Lgr5-EGFP^+^ retinal cells *in vivo*, we adapted a genetic lineage tracing approach. We further marked these postnatally born Lgr5^+^ amacrine cells with the *Rosa26-LacZ* reporter in the *Lgr5*^*EGFP-Ires-CreERT2*^*; Rosa26-LacZ* mice by IP injection of tamoxifen into these mice at P3 to P4 or 4–6 weeks of age. We then determined whether new cells could be generated from these cells at later times. We speculated that if new cells could be generated from Lgr5^+^ amacrine cells, they should be able to migrate to new retinal locations to replace damaged or lost cells. The highly organized stereotypical structure of the mammalian retina provides an advantage for this assay. Histochemical analysis of retina cross sections revealed that activation of the LacZ reporter at P3 to P4 resulted in X-gal staining that was restricted primarily to the second and third rows of inner nuclear layer cells in the retina of 3- to 4-week-old mice where the X-gal signal overlapped with the Lgr5-EGFP signal (Fig.3A). When analyzed at 2 months of age, LacZ^+^ cells were observed in the outer half of the inner nuclear layer and in cells localized to the retinal ganglion cell layer (Fig.3B). Similarly, when tamoxifen injections were given to 4- to 6-week-old mice, LacZ^+^ cells were detected only in Lgr5-EGFP^+^ cells in the inner half of the inner nuclear layer 2 weeks after injection (data not shown). However, when the mice were 6 months of age, LacZ^+^ cells were also detected at the outer edge of the inner nuclear layer where horizontal cells reside (Fig.3C). The presence of LacZ^+^ cells in new locations at later times supports the notion that Lgr5-EGFP^+^ amacrine cells may possess the capacity to regenerate new retinal cells.

To quantitate the frequency of new cell generation from Lgr5^+^ amacrine cells, we injected tamoxifen into 1-month-old *Lgr5*^*EGFP-Ires-CreERT2*^*; Rosa26-LacZ* mice and then measured the number of LacZ^+^ cells that had migrated to the ganglion cell layer and the percentage of LacZ^+^ cells present in the outer half of the inner nuclear layer as a function of time (Fig.3D–I). We observed that on flat-mount retina samples, approximately 50 LacZ^+^ cells on average had migrated to the ganglion cell layer of one retina 6 months after tamoxifen injection, and the number increased to over 100 one year later (Fig.3H). The frequency of new LacZ^+^ cell generation was much higher in the inner nuclear layer, with 0.9% and 2.1% of total LacZ^+^ cells being present in the outer half of the inner nuclear layer 1 and 5 months after tamoxifen injection (Fig.3I).

The detection of LacZ^+^ cells in new locations at later times could also be caused by tamoxifen-independent activation of the reporter in other retinal cell types as the mice mature. However, we failed to detect any leaked expression of the LacZ reporter in the retina of 6-month-old *Lgr5*^*EGFP-Ires-CreERT2*^*; Rosa26-LacZ* mice using X-gal staining (Fig. S4A–C; Supporting information). In addition, we observed LacZ-positive but Lgr5-EGFP-negative cells in the inner nuclear layer of flat-mount retina samples from animals that had received tamoxifen injections (Fig. S4D–J), further supporting the notion that LacZ^+^ cells observed in new locations were derived from Lgr5-EGFP^+^ retinal cells. Together, these results suggest Lgr5^+^ amacrine cells possess the potential to generate new retinal cells under normal physiological conditions. Newly generated cells first appear near Lgr5-EGFP^+^ cells and then migrate to other locations.

**Fig 4:**
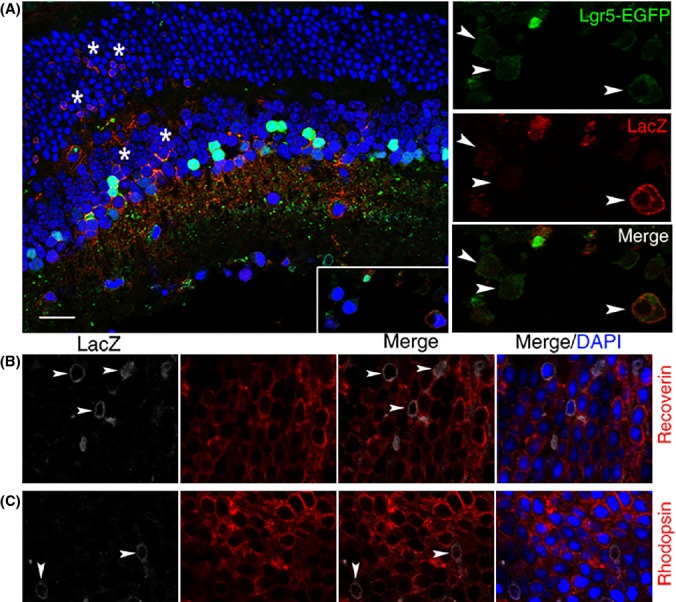
Generation of new retinal cells in response to retinal injury. (A) Confocal images of a retinal cross section from *Lgr5*^*EGFP*^^*-Ires-Cre*^^*ERT*^^*2*^*; Rosa26-LacZ* mice analyzed with anti-β-galactosidase (LacZ) antibody (red) following retinal NMDA and growth factor injection. LacZ-positive cells are present in all three cell layers of the retina (indicated by stars in the inner nuclear layer and outer nuclear layer). The adjacent higher magnification views show co-localization of the Lgr5-EGFP signal with LacZ in cells of the loosely organized retinal ganglion layer (highlighted with arrowheads). (B) Co-staining of LacZ with recoverin (a marker for photoreceptors). Arrowhead marks LacZ and recoverin double-positive photoreceptor cells. (C) Co-staining of LacZ with rhodopsin (a marker for rod photoreceptors). Arrowheads mark LacZ and rhodopsin double-positive photoreceptor cells. Scale bars = 20 μm.

We also examined the ability of Lgr5-EGFP^+^ cells to generate other retinal cells in a model of damage-induced retinal repair. *Lgr5*^*EGFP-Ires-CreERT2*^*; Rosa26-LacZ* pups were injected with tamoxifen twice at P3 and P4. At 3 months, the mice were given an ocular injection of the excitotoxin NMDA followed by a second injection of growth factors 2 days later. After two more days, mice were sacrificed and histochemical analysis was performed to examine the localization of LacZ^+^ cells. We observed that in response to injury, LacZ^+^ cells could be detected in all three retinal cell layers (Fig.4A), including photoreceptors of the outer nuclear layer (Fig.4B,C). Together, these results suggest that Lgr5-EGFP^+^ amacrine cells possess the capacity to generate other retinal cell types in adult mice.

### Lgr5^+^ amacrine cells contribute to retinal regeneration throughout life

**Fig 5:**
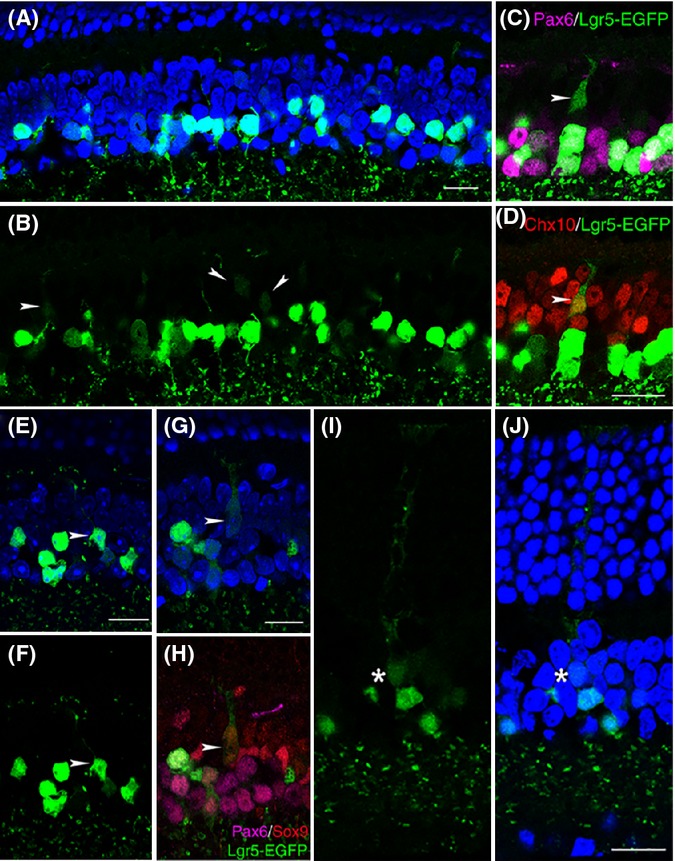
Generation of retinal neurons and Müller glial cells by Lgr5-EGFP^+^ amacrine cells in the retina under normal physiological conditions. (A, B) Confocal images of strong and weak Lgr5-EGFP-expressing cells in the inner nuclear layer of 6-week-old *Lgr5*^*EGFP*^^*-Ires-Cre*^^*ERT*^^*2*^ mice. Weakly Lgr5-EGFP-expressing cells are marked with arrowheads. (C, D) Dual immunohistochemical analysis of Lgr5-EGFP with Pax6 (C) and Lgr5-EGFP with Chx10 (D). In these views, the arrowhead marks a weak Lgr5-EGFP expression immature bipolar cell that lost Pax6 expression and was positive for Chx10 staining. (E, F) A Lgr5-EGFP^+^ cell with typical mature bipolar cell morphology, highlighted with arrowheads. (G, H) A weak Lgr5-EGFP-expressing immature Müller cell. In triple stainings of panel H, the weak Lgr5-EGFP-expressing immature Müller cell was negative for Pax6 staining, but positive for Sox9 staining. (I, J) A weakly Lgr5-EGFP-expressing mature Müller cell in the retina of a 15-month-old mouse. The star marks the cell body of the weakly Lgr5-EGFP-expressing cell. Nuclei are stained by DAPI (blue) in panels A, E, G, and J. Scale bars = 20 μm.

Using the Lgr5-EGFP marker, we next investigated the role of Lgr5-EGFP^+^ amacrine cells in retinal homeostasis. We reasoned that if Lgr5-EGFP^+^ retinal cells generate other retinal cells through dedifferentiation or transdifferentiation, some immediate progeny of Lgr5-EGFP^+^ cells should retain a weak EGFP signal after the Lgr5-EGFP gene has turned off, as it takes time for the EGFP protein to be degraded in these cells. In agreement with this thought, we observed that in 6-week-old mice, in addition to the presence of strong Lgr5-EGFP-expressing cells in the innermost three rows of cells in the inner nuclear layer, weak Lgr5-EGFP-expressing cells were also present in the middle-to-outer half of the inner nuclear layer (Fig.5A,B). Unlike strongly Lgr5-EGFP-expressing cells, weakly Lgr5-EGFP-expressing cells displayed projections emanating toward the outer plexiform layer. Based on their localization, morphology and gene expression, weakly Lgr5-EGFP-expressing cells were divided into two subgroups. One subgroup localized to the outer half of the inner nuclear layer, had thin projections and was positive for Chx10, but negative for Pax6 staining, suggesting these cells may represent early-stage bipolar cells (Fig.5C,D). By 2 months of age, these cells displayed bipolar cell morphology with prominent axonal projections toward the inner edge of the outer plexiform layer (Fig.5E,F). The second subgroup localized to the middle of the inner nuclear layer, had strong projections and was negative for Pax6 but positive for Sox9, suggesting they may represent immature Müller cells (Fig.5G,H). In older mice, these cells extended axonal processes to the outer edge of the outer nuclear layer, exhibiting a unique Müller cell morphology (Fig.5I,J). Generation of new bipolar cells and Müller cells from Lgr5-EGFP^+^ cells continued as the animal aged. We estimated that <0.5% of Lgr5-EGFP^+^ cells exhibited Müller cell morphology in both 2-month- and 15-month-old mice. By contrast, the percentage of Lgr5-EGFP^+^ cells that exhibited bipolar cell morphology increased from 0.91 ± 0.18% in 2-month-old mice to 4.27 ± 0.56% in 15-month-old mice (*n* = 3838 and 3603 cells counted from each group). The existence of these faint Lgr5-EGFP^+^ early-stage neuronal and glial cells and their transition to mature cells in the retina suggest that new cells could be generated from Lgr5-EGFP^+^ cells in the retina under normal physiological conditions.

**Fig 6:**
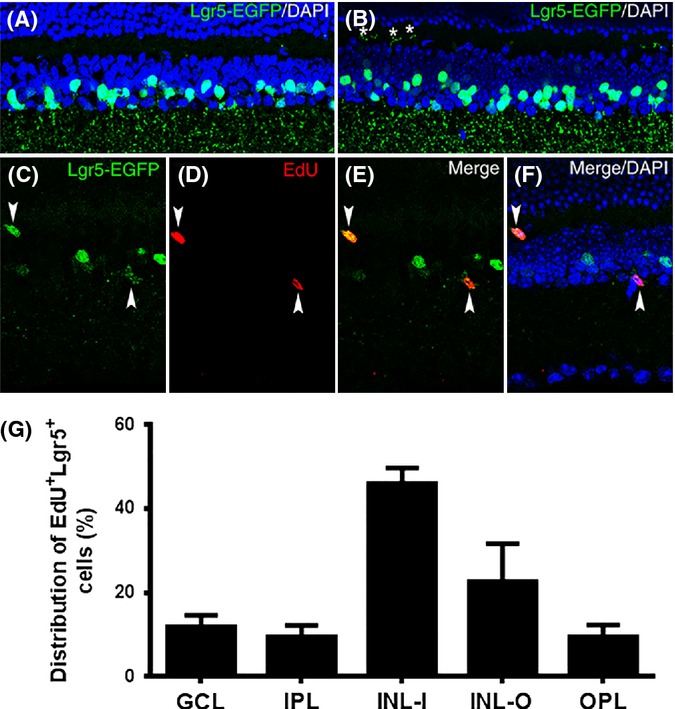
Labeling of Lgr5-EGFP^+^ cells with EdU in the retina of adult mice. (A, B) Confocal images of the retina obtained from a 2-month-old *Lgr5*^*EGFP*^^*-Ires-Cre*^^*ERT*^^*2*^ mouse (A) and an 8-month-old *Lgr5*^*EGFP*^^*-Ires-Cre*^^*ERT*^^*2*^ mouse (B). Stars in panel B highlight the processes of some Lgr5-EGFP^+^ cells that project into the outer plexiform layer at this age. (C–F) Confocal images of the retina from a 2.5-month-old *Lgr5*^*EGFP*^^*-Ires-Cre*^^*ERT*^^*2*^ mouse labeled with EdU. EdU and Lgr5-EGFP double-positive cells are highlighted with arrowheads. (G) Distribution of EdU and Lgr5-EGFP double-positive cells across the retina. *n* = 62 sections from three mice. GCL, ganglion cell layer; IPL, inner plexiform layer; INL-I, inner half of inner nuclear layer; INL-O, outer half of the inner nuclear layer; OPL, outer plexiform layer; ONL, outer nuclear layer.

Although new cells are continuously generated from Lgr5-EGFP^+^ cells in the retina of adult mice, the abundance of Lgr5-EGFP^+^ cells does not obviously decline with age (Fig.6A,B), suggesting that Lgr5-EGFP^+^ cells might be able to proliferate *in vivo*. To test this hypothesis, we injected the thymidine analog EdU into adult *Lgr5*^*EGFP-Ires-CreERT2*^ mice to detect *de novo* DNA synthesis in Lgr5-EGFP^+^ amacrine cells. We observed that some Lgr5-EGFP^+^ cells could be labeled with EdU (Fig.6C–G), suggesting that these cells may be able to reenter the cell cycle under certain circumstances. Furthermore, Lgr5-EGFP^+^EdU^+^ cells were found in both young (2 months old) and aged (15 months old) mice. Taken together, these results suggest Lgr5^+^ retinal cells possess regenerative capacity and may contribute to retinal homeostatic maintenance throughout the lifetime of the animal.

## Discussion

The generation of new retinal neurons and glia by Lgr5-EGFP^+^ amacrine interneurons in adult mice was surprising, as the current dogma states that differentiated neurons are postmitotic and represent a developmental endpoint. To identify these regenerative retinal cells, we adapted a genetic strategy based on the Lgr5-EGFP reporter, which was used successfully to identify adult stem cells in other tissues (Barker *et al*., 2007; Snippert *et al*., 2010; Chai *et al*., 2012; Shi *et al*., 2012; Yee *et al*., 2013; Chen *et al*., 2014). Here, we found that Lgr5^+^ retinal cells were localized to the inner nuclear layer, projected axonal processes to the inner plexiform layer, and expressed markers typical of amacrine interneurons (Fig.1, and Figs S2 and S3). Surprisingly, however, genetic lineage tracing demonstrated that some of these cells may still possess regenerative capacity and function as an endogenous regenerative source in adult mice.

Although it is widely accepted that stem cells should lack features attributed to more mature cells, accumulating evidence shows that adult stem cells frequently possess characteristics of differentiated cells. For example, stem cells in the subventricular zone of the lateral ventricle and the subgranular layer of the hippocampal dentate gyrus in the adult brain display characteristics of differentiated astrocytes (Doetsch *et al*., 1999; Seri *et al*., 2001; Zhao *et al*., 2008). Similarly, multipotent stem cells in mouse and human endocrine pancreas express insulin (Smukler *et al*., 2011). The highly specialized anatomy of the mammalian retina precludes the support of a highly proliferative stem cell population. Rather, cells that serve as the source of retinal renewal are unlikely to be highly proliferative under normal physiological conditions and may have additional functions. In accord with this idea, the regenerative Lgr5^+^ retinal cell identified here also possess features of differentiated neurons.

Ontogenic analyses uncovered two populations of temporally regulated Lgr5^+^ cells in the mouse retina. The first *Lgr5*^+^ population was transiently expressed in the early stages of embryonic retinogenesis. *Lgr5* expression in the embryonic retina was silenced before E15 and did not reappear until P2, when it was expressed specifically in differentiated late-born amacrine interneurons and persisted in these cells throughout the animal’s life. The postnatal expression of Lgr5 in differentiated amacrine cells was recently confirmed by another independent study, which did not investigate the embryonic expression of *Lgr5* (Sukhdeo *et al*., 2014). We found that although the embryonic *Lgr5* expression was transient, it was nonetheless essential for ocular development as newborn Lgr5^−/−^ pups displayed a dramatic reduction in eye size (data not shown). These findings are consistent with previous studies showing that the majority of early-stage embryonic retinal cells are retinal progenitors, and suggest that Lgr5^+^ retinal progenitor cells can generate multiple retinal cell lineages. Subsequently, *Lgr5* expression reappeared postnatally in a subset of amacrine interneurons that, while possessing characteristics of differentiated cells, were nonetheless able to contribute to regeneration in adult mammalian retina. Interestingly, an earlier report suggested that retinal stem cells were localized to the ciliary body (Tropepe *et al*., 2000). These cells, however, were subsequently identified as pigmented epithelial cells, which could form clonogenic spheres but failed to differentiate into retinal neurons (Cicero *et al*., 2009; Gualdoni *et al*., 2010). Our finding that Lgr5-EGFP expression was restricted to the neural retina and was absent in other ocular regions, including the ciliary body, supports this conclusion (Fig. S1).

Two mechanisms may contribute to Lgr5^+^ amacrine cell-medicated retinal regeneration. Lgr5^+^ amacrine cells could directly transdifferentiate into other retinal cells by turning off the *Lgr5* gene and regaining expression of genes essential for new regenerated cells. Alternatively, Lgr5^+^ amacrine cells could first dedifferentiate into a proliferative stem or progenitor cell-like status and then differentiate into new retinal cells. The detection of Lgr5-EGFP^+^EdU^+^ cells in the adult retina (Fig.6C–F) supports the possibility of Lgr5^+^ amacrine cell dedifferentiation. Consistent with this, many of Lgr5-EGFP^+^EdU^+^ cells have migrated to new locations, suggesting that they may have dedifferentiated (Fig.6G). In addition, some newly generated LacZ^+^ cells in the ganglion cell layer occasionally form clusters (Fig.3F), suggesting that they might be derived from a single precursor cell. However, the frequency of Lgr5-EGFP^+^EdU^+^ cells in the retina is very low even with multiple injections of this thymidine analog (94 cells in 62 retina sections). This may have contributed to previous failure in identifying similar cells with thymidine analogs in the adult mammalian retina.

Lgr5 is a G-protein-coupled receptor. Its ligands have recently been identified as R-spondins, a group of secreted proteins (Carmon *et al*., 2011; de Lau *et al*., 2011). Binding of R-spondins to Lgr5 triggers the interaction of Lgr5 with Wnt receptors to modulate the Wnt/β-catenin signaling pathway (Carmon *et al*., 2012). Although the Wnt/β-catenin signaling pathway has been well characterized in stem cell self-renewal and differentiation in high-turnover epithelial tissues, its function in the Lgr5^+^ amacrine cell population is unknown. It would be interesting to investigate how this signaling pathway regulates Lgr5^+^ amacrine cell differentiation and retinal homeostatic maintenance in the future.

Reminiscent of our findings in mice, fish express two types of retinal stem cells (Cameron & Easter, 1995; Otteson *et al*., 2001; Wu *et al*., 2001; Centanin *et al*., 2011). Ciliary marginal zone retinal stem cells continually add new cells to the periphery of the existing retina. However, the ciliary marginal zone in mammals is evolutionarily lost, precluding this tissue from serving as a viable stem cell reservoir. Fish additionally express a second group of retinal stem cells that form clusters in the inner nuclear layer. These cells proliferate and migrate to the outer nuclear layer, ultimately generating new photoreceptors. Similar to the mouse retinal Lgr5^+^ cells reported here, the second group of fish retinal stem cells express Pax6 and proliferate slowly as they can only be detected by long-term BrdU or ^3^H-thymidine administration (Otteson *et al*., 2001). However, these fish cells were later identified as Müller glial cells (Bernardos *et al*., 2007). The parallels between the two stem cell populations present in the fish retina and the embryonic and postnatal Lgr5^+^ cells in the mouse retina are striking. It will be of interest to determine whether these fish cells also have neuronal features and their evolutionary relationship with Lgr5^+^ mammalian amacrine cells in future efforts.

In summary, the identification of Lgr5^+^ amacrine cells in the adult mammalian retina that may possess regenerative capacity provides new opportunities to study neuronal regeneration in mammals. These findings may also provide insight into new therapies for the treatment of blindness in humans. Indeed, irreversible neuronal loss in the retina is a leading cause of vision impairment in the aged population. The identification of Lgr5^+^ adult retinal cells with regenerative capacity reported here may therefore provide new approaches to treat age-related retinal degenerative diseases in patients.

## Experimental procedures

### Animals

*Lgr5*^*EGFP-Ires-CreERT2*^ knock-in mice (Barker *et al*., 2007) and Rosa26-LacZ mice were obtained from the Jackson Laboratory. These two mouse strains were crossed to generate the *Lgr5*^*EGFP-Ires-CreERT2*^*; Rosa26-LacZ* mice. All animals were housed in a facility with a 12-h light/12-h dark cycle. All animal experiment procedures were conducted with both male and female mice and were approved by the Animal Care and Use Committee of the University of Pittsburgh.

### Histology and immunostainings

Mice were euthanized with CO_2_ followed by cervical dislocation. Dissected mouse eyeballs were then enucleated and fixed with 4% paraformaldehyde in 1× PBS overnight at 4 °C. After being equilibrated in a sucrose gradient (5, 15, and 30%) in 1× PBS and then a 1:1 mixed solution of 30% sucrose and optimal cutting temperature compound, samples were embedded in optimal cutting temperature compound and frozen quickly on dry ice. Retinal cryosections were cut (10 μm) using an HM Cryostat (Thermo Scientific, Waltham, MA, USA).

Retinal sections were blocked with 0.5% BSA supplemented with 5% serum of the host animal from which the secondary antibody was derived. Primary antibodies used in this study were as follows: rabbit anti-GAD (Millipore, Billerica, MA, USA), goat anti-Glyt1 (Millipore, Billerica, MA, USA), rabbit anti-Sox9 (Millipore, Billerica, MA, USA), mouse anti-Pax6 (R&D system, Minneapolis, MN, USA), sheep anti-Chx10 (Millipore, Billerica, MA, USA), mouse anti-PKCα (Santa Cruz Biotechnology, Dallas, TX, USA), mouse anti-P27 (BD Biosciences, San Jose, CA, USA), mouse anti-CRALBP (Abcam, Cambridge, MA, USA), rabbit anti-calbindin (Abcam, Cambridge, MA, USA), rabbit anti-calretinin (Millipore, Billerica, MA, USA), mouse anti-syntaxin-1A (Sigma, St. Louis, MO, USA), rabbit anti-glutamine synthetase (Sigma, St. Louis, MO, USA), rabbit anti-recoverin (Chemicon, Temecula, CA, USA), PNA Alexa Fluor Conjugate (Life Technologies, Grand Island, NY, USA), rat anti-Thy1.2 (Abcam, Cambridge, MA, USA), mouse anti-beta-galactosidase (Promega, Madison, WI, USA), and rabbit anti-beta-galactosidase (Abcam, Cambridge, MA, USA). The specificity of all primary antibodies was tested in pilot experiments.

### Microscopy

Immunofluorescence confocal images were acquired using an Olympus confocal microscope (Tokyo, Japan). Images were processed and analyzed using FV1000 Viewer software (Tokyo, Japan) with minor adjustments for brightness and contrast.

### X-gal staining

After brief fixation in 4% paraformaldehyde and 0.2% glutaraldehyde in 1× PBS, retinal cryosections were incubated in the X-gal staining solution [0.1% X-gal, 2 mm MgCl_2_, 5 mm EGTA, 0.02% Nonidet P-40, 5 mm K_3_Fe(CN)_6_, and 5 mm K_4_Fe(CN)_6_.6H_2_O] overnight at 35 °C. After staining, sections were counterstained with Nuclear Fast Red and then dehydrated through an ethanol gradient. Slides were then mounted with Permount medium and imaged using an Axion Observer Inverted Microscope (Carl Zeiss Microscopy, Bovenden, Germany).

### Intraocular injections of NMDA and growth factors

Mice were anesthetized by IP injection of Avertin at a dosage of 250–500 mg kg^−1^ body weight. Eyes were injected with 1.5 μL of 0.2 m NMDA with a glass micropipette injector. Two days after NMDA injection, the same eye was injected with 2 μL of a mixture of insulin (3 μg μL^−1^) and FGF (10 ng mL^−1^). Animals were sacrificed at the indicated time points after injection.

### *In vivo* EdU labeling of cell proliferation

The 6-week-old *Lgr5*^*EGFP-Ires-CreERT2*^ mice were IP-injected with EdU (50 mg kg^−1^ body weight) 10 times, with a 3-day interval between each injection. Mice were sacrificed after the last injection. EdU signals on retinal cross sections were detected with an EdU detection kit (Invitrogen).

### Tamoxifen injection

For *in vivo* lineage tracing experiments, tamoxifen was IP-injected into *Lgr5*^*EGFP-Ires-CreERT2*^*; Rosa26-LacZ* mice to activate the LacZ reporter gene. P3 to P4 pups received two injections of tamoxifen (150 μg in 20 μL of corn oil). Adult (4–6 weeks old) mice received 5 tamoxifen injections once the other day at a dose of 75 mg kg^−1^ body weight.

### Statistical analysis

For both *in vitro* and *in vivo* experiments, each experiment was repeated at least three times. Quantitative data were presented as mean ± SEM. When comparisons of two groups were presented, two-tailed *t*-tests were performed. *P* values <0.05 were considered to be statistically significant.

## Supporting Information

**Fig. S1**Lgr5-EGFP labels a population of inner nuclear layer cells in the retina of adult mice. **Fig. S2** Lgr5-EGFP^+^ cells are amacrine cells. **Fig. S3** Lgr5-EGFP^+^ amacrine cells project processes into the inner plexiform layer (IPL) and form synapses before the completion of retinogenesis. **Fig. S4** X-gal and LacZ stainings of whole-mount retinal samples.

## References

[b1] Ahmad I, Tang L, Pham H (2000). Identification of neural progenitors in the adult mammalian eye. Biochem. Biophys. Res. Commun.

[b2] Amato MA, Arnault E, Perron M (2004). Retinal stem cells in vertebrates: parallels and divergences. Int. J. Dev. Biol.

[b3] Barker N, van Es JH, Kuipers J, Kujala P, van den Born M, Cozijnsen M, Haegebarth A, Korving J, Begthel H, Peters PJ, Clevers H (2007). Identification of stem cells in small intestine and colon by marker gene Lgr5. Nature.

[b4] Bernardos RL, Barthel LK, Meyers JR, Raymond PA (2007). Late-stage neuronal progenitors in the retina are radial Muller glia that function as retinal stem cells. J. Neurosci.

[b5] Bhatia B, Singhal S, Jayaram H, Khaw PT, Limb GA (2010). Adult retinal stem cells revisited. Open Ophthalmol. J.

[b6] Cameron DA, Easter SS (1995). Cone photoreceptor regeneration in adult fish retina: phenotypic determination and mosaic pattern formation. J. Neurosci.

[b7] Carmon KS, Gong X, Lin Q, Thomas A, Liu Q (2011). R-spondins function as ligands of the orphan receptors LGR4 and LGR5 to regulate Wnt/beta-catenin signaling. Proc. Natl Acad. Sci. USA.

[b8] Carmon KS, Lin Q, Gong X, Thomas A, Liu Q (2012). LGR5 interacts and cointernalizes with Wnt receptors to modulate Wnt/beta-catenin signaling. Mol. Cell. Biol.

[b9] Centanin L, Hoeckendorf B, Wittbrodt J (2011). Fate restriction and multipotency in retinal stem cells. Cell Stem Cell.

[b10] Chai R, Kuo B, Wang T, Liaw EJ, Xia A, Jan TA, Liu Z, Taketo MM, Oghalai JS, Nusse R (2012). Wnt signaling induces proliferation of sensory precursors in the postnatal mouse cochlea. Proc. Natl Acad. Sci. USA.

[b11] Chen M, Tian S, Yang X, Lane AP, Reed RR, Liu H (2014). Wnt-responsive Lgr5(+) globose basal cells function as multipotent olfactory epithelium progenitor cells. J. Neurosci.

[b12] Cicero SA, Johnson D, Reyntjens S, Frase S, Connell S, Chow LM, Baker SJ, Sorrentino BP, Dyer MA (2009). Cells previously identified as retinal stem cells are pigmented ciliary epithelial cells. Proc. Natl Acad. Sci. USA.

[b13] Doetsch F, Caille I, Lim DA, Garcia-Verdugo JM, Alvarez-Buylla A (1999). Subventricular zone astrocytes are neural stem cells in the adult mammalian brain. Cell.

[b14] Dyer MA, Cepko CL (2000). Control of Muller glial cell proliferation and activation following retinal injury. Nat. Neurosci.

[b15] Fischer AJ, Reh TA (2001). Muller glia are a potential source of neural regeneration in the postnatal chicken retina. Nat. Neurosci.

[b16] van der Flier LG, Clevers H (2009). Stem cells, self-renewal, and differentiation in the intestinal epithelium. Annu. Rev. Physiol.

[b17] Gualdoni S, Baron M, Lakowski J, Decembrini S, Smith AJ, Pearson RA, Ali RR, Sowden JC (2010). Adult ciliary epithelial cells, previously identified as retinal stem cells with potential for retinal repair, fail to differentiate into new rod photoreceptors. Stem Cells.

[b18] Hsueh HA, Molnar A, Werblin FS (2008). Amacrine-to-amacrine cell inhibition in the rabbit retina. J. Neurophysiol.

[b19] Karl MO, Hayes S, Nelson BR, Tan K, Buckingham B, Reh TA (2008). Stimulation of neural regeneration in the mouse retina. Proc. Natl Acad. Sci. USA.

[b20] Kubota R, Hokoc JN, Moshiri A, McGuire C, Reh TA (2002). A comparative study of neurogenesis in the retinal ciliary marginal zone of homeothermic vertebrates. Brain Res. Dev. Brain Res.

[b21] Lamba D, Karl M, Reh T (2008). Neural regeneration and cell replacement: a view from the eye. Cell Stem Cell.

[b22] de Lau W, Barker N, Low TY, Koo BK, Li VS, Teunissen H, Kujala P, Haegebarth A, Peters PJ, van de Wetering M (2011). Lgr5 homologues associate with Wnt receptors and mediate R-spondin signalling. Nature.

[b23] Livesey FJ, Cepko CL (2001). Vertebrate neural cell-fate determination: lessons from the retina. Nat. Rev. Neurosci.

[b24] Luo CW, Hsueh AJ (2006). Genomic analyses of the evolution of LGR genes. Chang Gung Med. J.

[b25] MacNeil MA, Masland RH (1998). Extreme diversity among amacrine cells: implications for function. Neuron.

[b26] Masland RH (2001). The fundamental plan of the retina. Nat. Neurosci.

[b27] Moore DL, Goldberg JL (2010). Four steps to optic nerve regeneration. J. Neuroophthalmol.

[b28] Moshiri A, Close J, Reh TA (2004). Retinal stem cells and regeneration. Int. J. Dev. Biol.

[b29] Ooto S, Akagi T, Kageyama R, Akita J, Mandai M, Honda Y, Takahashi M (2004). Potential for neural regeneration after neurotoxic injury in the adult mammalian retina. Proc. Natl Acad. Sci. USA.

[b30] Osakada F, Ooto S, Akagi T, Mandai M, Akaike A, Takahashi M (2007). Wnt signaling promotes regeneration in the retina of adult mammals. J. Neurosci.

[b31] Otteson DC, D’Costa AR, Hitchcock PF (2001). Putative stem cells and the lineage of rod photoreceptors in the mature retina of the goldfish. Dev. Biol.

[b32] Pei YF, Rhodin JA (1970). The prenatal development of the mouse eye. Anat. Rec.

[b33] Rattner A, Nathans J (2006). Macular degeneration: recent advances and therapeutic opportunities. Nat. Rev. Neurosci.

[b34] Seri B, Garcia-Verdugo JM, McEwen BS, Alvarez-Buylla A (2001). Astrocytes give rise to new neurons in the adult mammalian hippocampus. J. Neurosci.

[b35] Shi F, Kempfle JS, Edge AS (2012). Wnt-responsive lgr5-expressing stem cells are hair cell progenitors in the cochlea. J. Neurosci.

[b36] Smukler SR, Arntfield ME, Razavi R, Bikopoulos G, Karpowicz P, Seaberg R, Dai F, Lee S, Ahrens R, Fraser PE (2011). The adult mouse and human pancreas contain rare multipotent stem cells that express insulin. Cell Stem Cell.

[b37] Snippert HJ, Haegebarth A, Kasper M, Jaks V, van Es JH, Barker N, van de Wetering M, van den Born M, Begthel H, Vries RG (2010). Lgr6 marks stem cells in the hair follicle that generate all cell lineages of the skin. Science.

[b38] Sukhdeo K, Koch CE, Miller TE, Zhou H, Rivera M, Yan K, Cepko CL, Lathia JD, Rich JN (2014). The Lgr5 transgene is expressed specifically in glycinergic amacrine cells in the mouse retina. Exp. Eye Res.

[b39] Tropepe V, Coles BL, Chiasson BJ, Horsford DJ, Elia AJ, McInnes RR, van der Kooy D (2000). Retinal stem cells in the adult mammalian eye. Science.

[b40] Voinescu PE, Kay JN, Sanes JR (2009). Birthdays of retinal amacrine cell subtypes are systematically related to their molecular identity and soma position. J. Comp. Neurol.

[b41] Wohl SG, Schmeer CW, Isenmann S (2012). Neurogenic potential of stem/progenitor-like cells in the adult mammalian eye. Prog. Retin. Eye Res.

[b42] Wu DM, Schneiderman T, Burgett J, Gokhale P, Barthel L, Raymond PA (2001). Cones regenerate from retinal stem cells sequestered in the inner nuclear layer of adult goldfish retina. Invest. Ophthalmol. Vis. Sci.

[b43] Yee KK, Li Y, Redding KM, Iwatsuki K, Margolskee RF, Jiang P (2013). Lgr5-EGFP marks taste bud stem/progenitor cells in posterior tongue. Stem Cells.

[b44] Zhao C, Deng W, Gage FH (2008). Mechanisms and functional implications of adult neurogenesis. Cell.

