# Supplementary material for: Lgr5+ amacrine cells possess regenerative potential in the retina of adult mice
Source: Aging Cell. 2015 May 20;14(4):635–43. doi: 10.1111/acel.12346 (PMC4531077; doi:10.1111/acel.12346)
Supplement: Supplementary file 1 [file acel0014-0635-sd1.docx]

**SUPPORTING INFORMATION**

**
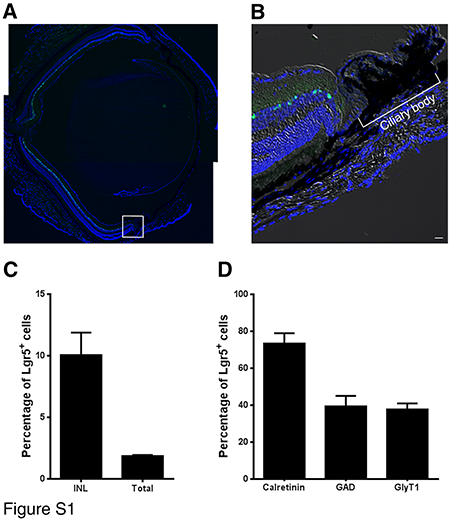
**

**Figure S1**. Lgr5-EGFP labels a population of inner nuclear layer cells in the retina of adult mice. (A) Low magnification view of Lgr5-EGFP expression in the retina of a 8-week old *Lgr5^EGFP-Ires-CreERT2^* mouse. (B) Higher magnification view of the boxed area in panel A. (C) Percentage of cells that are Lgr5-EGFP^+^ in the inner nuclear layer (INL) and in the retina (Total). (D) Percentage of Lgr5-EGFP^+^ cells that co-stain with specific retinal cell markers. Nuclei are stained by DAPI (blue). Lgr5-EGFP is in green. Scale bar, 30 µm.


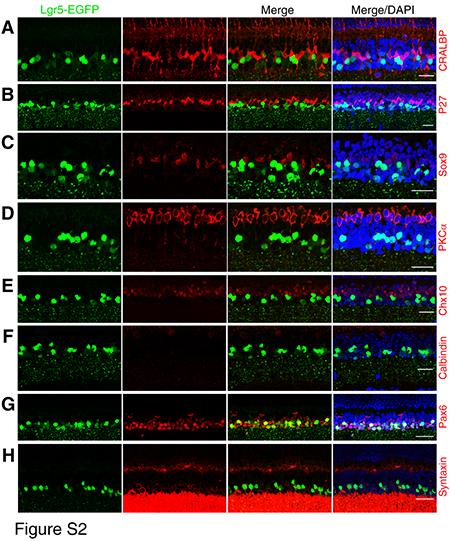


**Figure S2**. Lgr5-EGFP^+^ cells are amacrine cells. (A-F) Confocal images of retina from *Lgr5^EGFP-Ires-CreERT2^* mice stained with antibodies specific to Müller cells (CRLBP, P27, and Sox9 in A-C), bipolar cells (PKCα and Chx10 in D-E), and horizontal cells (Calbindin in F). (G-H) Lgr5-EGFP^+^ retinal cells express the transcription factor Pax6 (G) and stains with syntaxin (H). Scale bars, 30 µm.


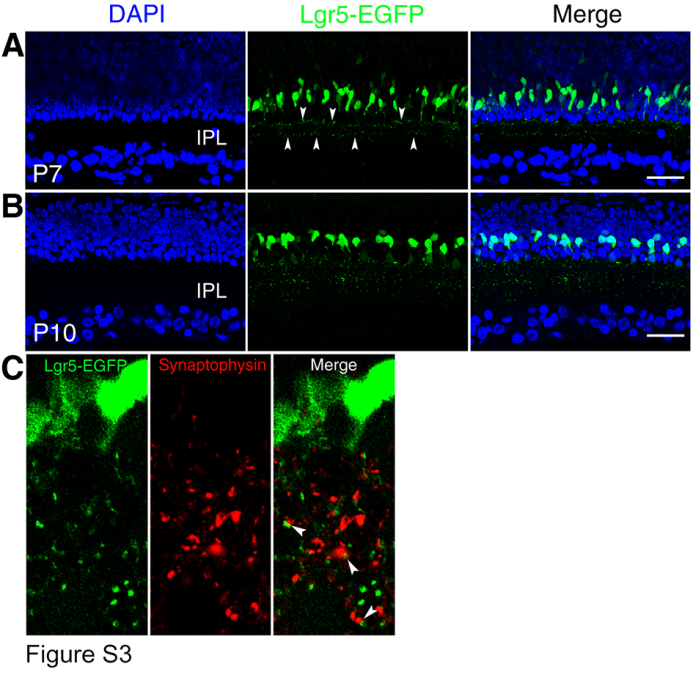


**Figure S3**. Lgr5-EGFP^+^ amacrine cells project processes into the inner plexiform layer (IPL) and form synapses before the completion of retinogenesis. (A) Confocal images of a retinal section from P7 *Lgr5^EGFP-Ires-CreERT2^* mice. Arrowheads highlight the incorporation of Lgr5-EGFP^+^ amacrine cell processes into loosely organized sublaminal structures. (B) Confocal images of a retinal section from P10 *Lgr5^EGFP-Ires-CreERT2^* mice. At this stage, processes of Lgr5-EGFP^+^ cells already form synapse-like structures. (C) Staining of the IPL of a P12 *Lgr5^EGFP-Ires-CreERT2^* mouse retina. Arrowheads highlight areas where Lgr5-EGFP^+^ synapse-like structures form close contacts with synatophysin positive presynapses. Scale bars, 30 µm.


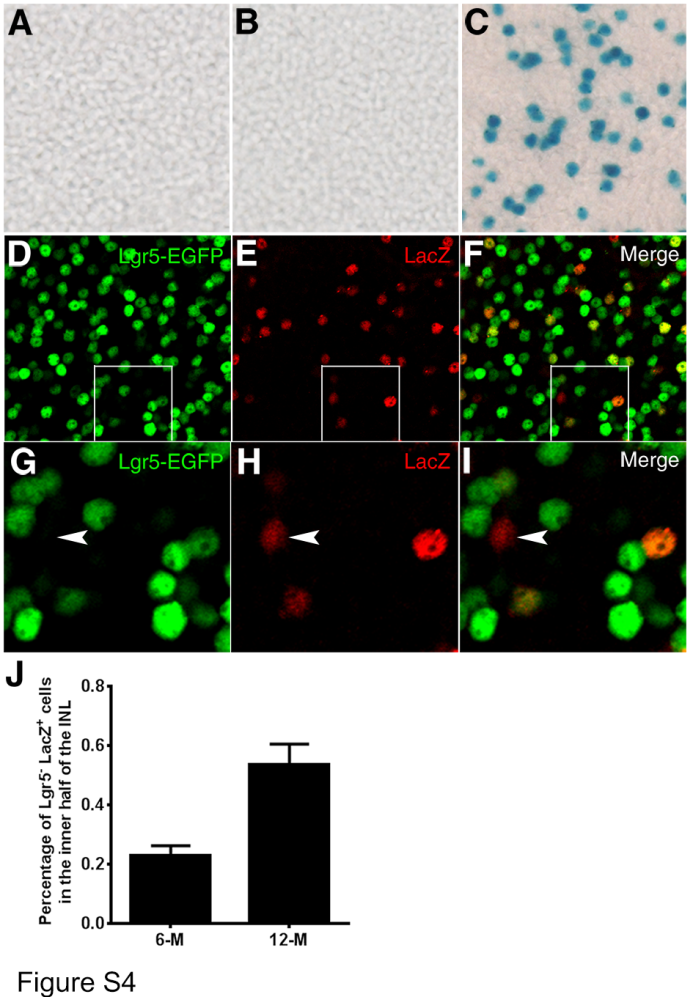


**Figure S4.** X-gal and LacZ stainings of whole-mount retinal samples. (A) Representative image of X-gal staining of a retina sample from a 6-month old *Lgr5^EGFP-Ires-CreERT2^; Rosa26-LacZ* -mouse that did not receive tamoxifen injection. No any X-gal positive cells are observed across the whole retina, indicating that there is no leaked expression of the Rosa26-LacZ reporter. Experiments were repeated over 3 times. (B-C) X-gal staining of retinal samples from adult *Lgr5^EGFP-Ires-CreERT2^; Rosa26-LacZ* mice that were sacrificed 2 days (*B*) or one month (C) after tamoxifen injections. Because of the presence of the blood-retina barrier, the efficiency of tamoxifen-induced LacZ activation in retinal Lgr5-EGFP^+^ cells islower than in other tissues. Only approximately 70% Lgr5-EGFP positive retinal cells were labeled with the LacZ reporter one month after tamoxifen injections. This further supports that LacZ positive cells observed in new locations where Lgr5-EGFP^+^ cells were not normally present were not caused by leaked expression of the LacZ reporter. (D-I) Confocal images of anti-β-galactosidase (LacZ) staining of whole-mount retina sample from *Lgr5^EGFP-Ires-CreERT2^; Rosa26-LacZ* mice that had received tamoxifen injection 12 months earlier. Boxed areas in panels D through F are highlighted with higher magnification in panels G through I. Arrowheads mark LacZ^+^ Lgr5-EGFP^-^ cells that were derived from Lgr5-EGFP^+^ cells but have turned off the *Lgr5-EGFP* transgene. (J) Percentage of LacZ^+^ Lgr5-EGFP^-^ cells in the inner half of the inner nuclear layer from 6-month old and 12-month old *Lgr5^EGFP-Ires-CreERT2^; Rosa26-LacZ* mice that had received tamoxifen injection at one month of age. n = 7 and 9 sections from 3 mice in each group. *P < 0.05.
